# Hypertonic dextrose prolotherapy and combined corticosteroid injection for lateral epicondylalgia: a comparative study

**DOI:** 10.3389/fpain.2026.1976214

**Published:** 2026-10-30

**Authors:** Xiao Li, Bin Huang, Guilian Huang, Zhaoxing Ou

**Affiliations:** 1 Department of Ultrasound, The Fifth People's Hospital of Zhuhai, Zhuhai, China; 2 Department of Ultrasound, The Second People's Hospital of Dingxi, Dingxi, China; 3 Department of Ultrasound, The Fifth Affiliated Hospital of Sun Yat-sen University, Zhuhai, China

**Keywords:** corticosteroid injection, lateral epicondylalgia, prolotherapy, tennis elbow, ultrasound-guided interventional therapy

## Abstract

**Background:**

Corticosteroid injection offered short-term efficacy for lateral epicondylalgia (LE) but limited long-term benefit, whereas hypertonic dextrose prolotherapy (HDPrT) yields superior medium-to-long-term outcomes. This study aimed to compare the efficacy of hypertonic dextrose prolotherapy combined with corticosteroid injection (HDPrT-C) versus HDPrT in the treatment of LE.

**Methods:**

Patients receiving ultrasound-guided HDPRT-C or HDPrT from May 2022 to December 2023 were retrospectively enrolled. HDPrT-C group received intratendinous injections of hypertonic dextrose and lidocaine, combined with peritendinous injections of corticosteroid and lidocaine. HDPrT group received only hypertonic dextrose and lidocaine. Patient assessments, including visual analogue scale (VAS) scores, tendon thickness, and tendon blood supply grade, were conducted at baseline, 1, 4, 12, and 24 weeks.

**Results:**

42 patients with LE were included, of whom 50% received HDPrT-C. HDPrT-C group significantly reduced the VAS scores and tendon thickness at short-term (1 and 4 weeks) follow-up compared to HDPrT (*P* < 0.05), while no statistically significant intergroup difference was observed in the improvement of tendon blood supply grade. At medium-term (12 weeks) follow-up, the tendon thickness in the HDPrT-C group was higher than that in the HDPrT group (mean difference, 0.2 mm; 95%CI: 0.09–0.39, *P* < 0.05). At long-term (24 weeks) follow-up, HDPrT-C group showed reductions in tendon thickness and improvements in tendon blood supply grade compared to HDPrT group (*P* < 0.05). No statistically significant intergroup difference was observed in the improvements of VAS scores at 12 weeks and 24 weeks.

**Conclusion:**

Compared with HDPrT, HDPrT C produced superior short term pain relief and greater reduction of tendon thickness in LE patients. Definitive medium- and long-term comparative efficacy warrant further investigation in larger trials.

## Introduction

Lateral epicondylalgia (LE), commonly known as tennis elbow, is a chronic injury and degenerative lesion of the common extensor tendon at its attachment point on the lateral humeral epicondyle (1). With a prevalence of 1%–3%, it predominantly affects individuals subjected to prolonged, high-load exertion of the forearm extensors, such as manual laborers (e.g., chefs, machine operators, programmers) and athletic populations (e.g., tennis or badminton players) (2). Clinical presentation typically includes lateral elbow pain, weakness, and limited mobility (1). The condition is often self-limiting, with symptoms frequently resolving within 6–12 months (3). The histopathological hallmarks of LE include fibroblast proliferation, collagen degeneration, and neovascularization (4). While local injection therapies, such as corticosteroids and hypertonic glucose, are commonly employed for LE refractory to conservative management, a consensus on optimal injectable agents remains elusive (5).

Corticosteroid injection (CSI) is the most frequently utilized non-surgical treatment for LE (6). Previous studies indicated that CSI exerted a substantial beneficial effect on all outcome measures for short-term pain relief (7). However, its medium- and long-term efficacy is contentious. Krogh et al. found no difference in pain relief between corticosteroid and placebo injections at 8 weeks (8). Coombes BK et al. reported that while CSI provided short-term benefit for tendinopathy, its efficacy at medium- and long-term follow-up might be inferior to other treatment modalities (9).

Hypertonic dextrose prolotherapy (HDPrT) was proved as a cost-effective, non-operative treatment for chronic musculoskeletal inflammation (10, 11). Its proposed mechanism of action involves local cellular dehydration and lysis induced by the hypertonic solution, leading to an influx of growth factors and inflammatory cells (12). This process was believed to initiate a localized injury-repair cascade to promote healing. *In vitro* studies suggested that cells exposed to dextrose proliferative therapy upregulate pro-inflammatory markers, including interleukin-8, cyclooxygenase-2, and prostaglandin E2, while downregulating the anti-inflammatory marker transforming growth factor-beta, indicating a potential mechanism for initiating the healing cascade (13). Pi et al. demonstrated that HDPrT was effective for medium- and long-term pain control, as well as for functional improvement in the mediate term (14). A meta-analysis further showed that the HDPrT group experienced an average reduction of 15.04 points in physical component scores compared to the control group at 12 weeks (11). Kaya et al. found HDPrT to yield superior patient outcomes compared to corticosteroid injection at 6-month follow-up (15). Nevertheless, HDPrT might offer less pronounced efficacy in the short term compared to CSI.

It remains unclear whether the combination of hypertonic glucose precursor therapy with corticosteroid injection (HDPrT-C) yields superior therapeutic outcomes compared to HDPrT alone. Therefore, this study aimed to investigate and compare the efficacy of HDPrT-C versus HDPrT in the treatment of LE.

## Methods

### Study subjects

This study retrospectively enrolled 48 patients diagnosed with LE at our center between May 2022 and December 2023, with a follow-up period of 24 weeks. This retrospective study was approved by the Ethics Committee of Zhuhai Fifth People's Hospital (No.2021093). The ethics committee waived informed consent for the retrospective use of clinical data. The study was performed in agreement with the Declaration of Helsinki. Two patients with a history of elbow trauma and four patients with rheumatoid arthritis were excluded, resulting in 42 patients being included in the final analysis. The study protocol was conducted in accordance with the Declaration of Helsinki. The study flowchart is presented in Figure 1.

**Figure 1:**
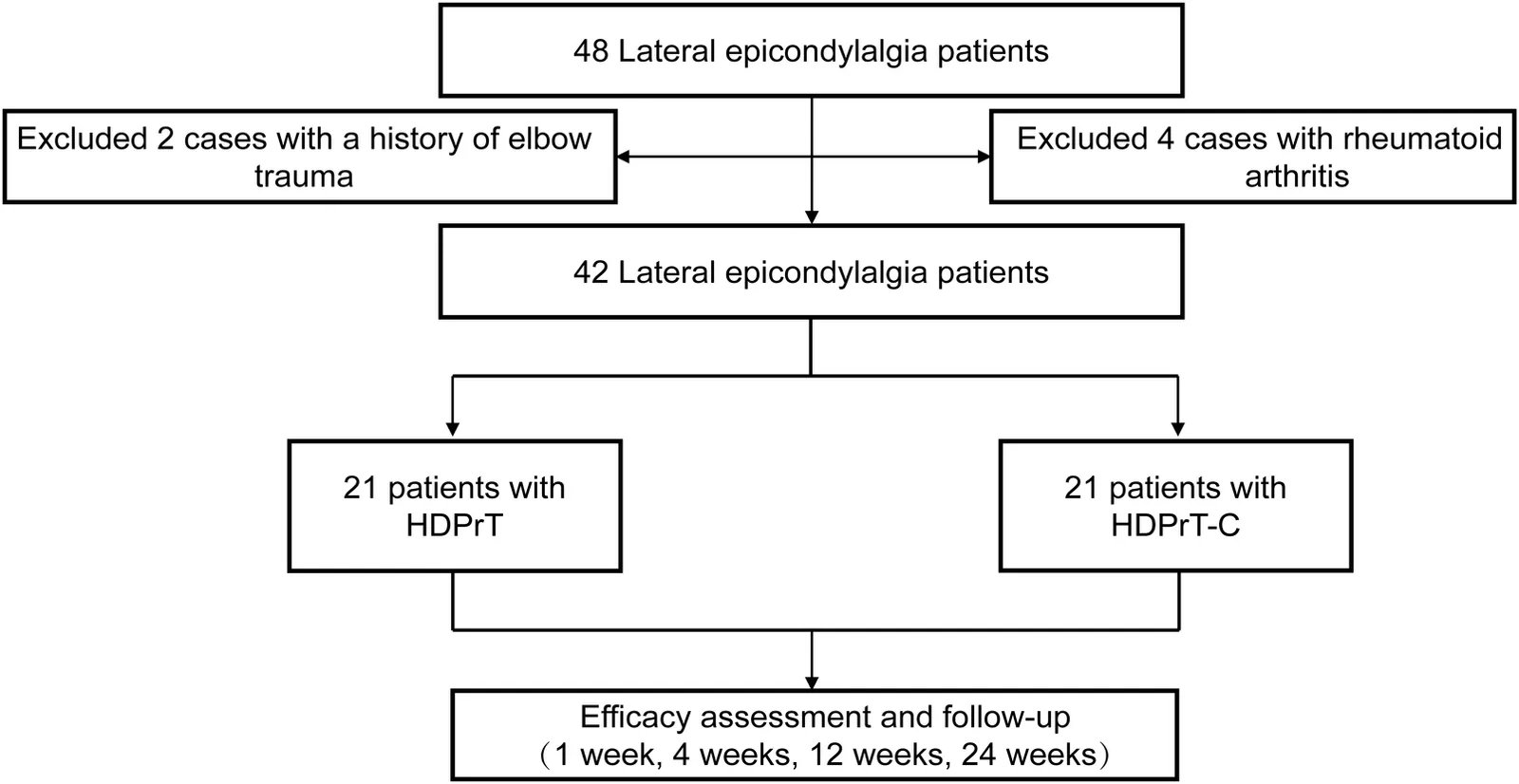
The workflow of the study. HDPrT: Hypertonic Dextrose Prolotherapy; HDPrT-C: Hypertonic Dextrose Prolotherapy Combined with Corticosteroid Injection.

Inclusion Criteria: 1) age ≥18 years, meeting the diagnostic criteria for LE (a history of pain for at least three months, tenderness upon palpation of the lateral epicondyle, and increased pain at the lateral epicondyle during resisted wrist extension) (1); 2) ultrasonographic findings of tendon thickness >5 mm or a tendon blood supply grade >1; 3) candidate for ultrasound-guided injection therapy.

Exclusion Criteria: 1) contraindications or history of allergy to corticosteroids; 2) history of elbow trauma or other inflammatory pathologies such as gouty arthritis or rheumatoid arthritis; 3) recent receipt of steroid injection therapy; 4) loss to follow-up.

### Treatment protocol

Injection Agents and Dosage: Compound betamethasone injection (1 mL); 50% glucose injection (10 g); 2% lidocaine injection (5 mL). A multi-point injection technique was employed.

HDPrT-C Group: 50% glucose injection (1.0 mL) mixed with 2% lidocaine (1.0 mL). Dose administered: 1.5 mL. Injection site: Intratendinous, targeting areas of rich blood supply.2% lidocaine (1.0 mL) mixed with compound betamethasone (0.5 mL). Injection site: Peritendinous.

HDPrT Group: 50% glucose injection (1.0 mL) mixed with 2% lidocaine (1.0 mL). Dose administered: 1.5 mL. Injection site: Intratendinous, targeting areas of rich blood supply.2% lidocaine (1.5 mL). Injection site: Peritendinous.

Ultrasound-guided injection was performed using a GE LOGIQ-E9 ultrasound system with a 6–15 MHz linear array transducer. The procedure was performed as follows: 1) the patient was placed in a supine position with the affected elbow slightly flexed and resting on the bed. The transducer was placed over the lateral humeral epicondyle; 2) sagittal, coronal, or oblique planes were used to identify the optimal view clearly displaying intratendinous hypoechoic areas and regions of rich blood supply, which served as the target for treatment; 3) the puncture site was marked under ultrasound guidance. Standard skin disinfection and draping were performed, followed by local anesthesia at the proximal mark; 4) while the transducer was stabilized with the left hand, the syringe was held in the right hand. The needle was inserted from the proximal end of the common extensor tendon using an ultrasound-guided in-plane approach. Upon reaching the intratendinous area of rich blood supply and confirming no aspiration of blood, the agent was injected at multiple points within the tendon and around its surface.

### Efficacy assessment and follow-up

Efficacy was evaluated using three primary outcome measures:

Visual Analogue Scale (VAS) scores (16): Pain intensity was assessed using a 100-mm horizontal line, where 0 mm represented “no pain” and 100 mm represented “the worst pain imaginable.” Higher scores indicated more severe pain.
Tendon Thickness: Measured in the sagittal plane approximately 5 mm distal to the bony cortex, from the bone surface to the superficial tendon border.
Tendon Blood Supply (graded using the Adler semiquantitative Doppler scale) (17): Grade 0: No detectable flow; Grade I: Minimal flow (1–2 dot or stick-like signals, with stick-like flow not exceeding half the lesion diameter); Grade II: Moderate flow (3–4 dot signals or one longer vessel penetrating near or beyond the radius); Grade III: Marked flow (≥5 dot signals or 2 longer vessels).

Follow-up assessments were conducted at 1 week, 4 weeks, 12 weeks, and 24 weeks post-treatment. VAS scores, tendon thickness, and tendon blood supply grade were recorded at baseline and each follow-up interval. Any adverse events or complications related to the injection procedure, such as localized swelling, increased pain, or aggravated limitation of elbow motion, were documented for both groups. VAS scores were collected via face-to-face clinic follow-up visits: patient self-completed the 100-mm paper visual analogue scale, and one outcome assessor transcribed the results into the research database. The assessor was blinded to LE patient treatment group. A single experienced sonographer completed all serial measurements across baseline and follow-up time-points, and was blinded to LE patient treatment group. All treatment procedures were performed by another experienced sonographer. No intra- or inter-observer reliability was performed.

### Statistical analysis

Data analysis was performed using SPSS software (version 17.0). Continuous variables with a normal distribution are expressed as mean ± standard deviation and were compared between groups using independent samples t-tests. Categorical data are presented as counts (percentages) and were compared using the Chi-square test or Fisher's exact test, as appropriate. A *P*-Value of less than 0.05 was considered statistically significant.

For continuous repeated-measure outcomes (VAS scores and tendon thickness), repeated-measures ANOVA was used to evaluate within-group changes over time, between-group effects, and group-by-time interaction. Mauchly's test of sphericity was performed. Greenhouse-Geisser correction was to be applied if the sphericity assumption was violated. Upon detection of a significant group-time interaction, *post-hoc* simple-effect analyses were performed to assess between-group differences at each follow-up time point and within-group changes relative to baseline. Bonferroni correction was applied for *post-hoc* pairwise comparisons to adjust for multiple testing. For within-group *post-hoc* pairwise comparisons across five time points (baseline, 1 week, 4 weeks, 12 weeks, and 24 weeks), four pairwise comparisons were conducted for each group. Bonferroni correction was calculated as *α*=0.05/4 = 0.0125, rounded to 0.012 as the adjusted significance threshold. *Post-hoc* pairwise comparisons were considered statistically significant when adjusted *P* < 0.012.

For the tendon blood supply grading, the non-parametric Friedman test was applied for within-group repeated-measures comparisons across follow-up time-points. When the overall Friedman test yielded statistical significance, *post-hoc* pairwise comparisons between individual time points were performed using paired-sample Wilcoxon signed-rank tests. Between-group comparisons at each time-point were conducted with the Mann–Whitney U test. For these multiple non-parametric *post-hoc* pairwise tests across time points, Bonferroni correction was implemented for these non-parametric *post-hoc* pairwise comparisons to account for multiple testing. For within-group non-parametric *post-hoc* pairwise comparisons across five time points (baseline, 1 week, 4 weeks, 12 weeks, and 24 weeks), four non-parametric pairwise comparisons were conducted for each group. Bonferroni correction was calculated as *α*=0.05/4 = 0.0125, rounded to 0.012 as the adjusted significance threshold. Non-parametric *post-hoc* pairwise comparisons were considered statistically significant when adjusted *P* < 0.012.

## Results

### Characteristics

A total of 42 patients were included in this study, comprising 25 females (59.52%), with a mean age of 49.00 ± 8.07 years. 21 patients (50.00%) were enrolled in the HDPrT-C group. No statistically significant differences were observed between the two groups regarding age, duration of pain (months), sex, affected side, baseline VAS score, baseline tendon thickness, or baseline blood supply grade (all *P* > 0.05). The general characteristics of patients in the HDPrT-C and HDPrT groups are presented in Table 1.

**Table 1:** Characteristic of patients in the two treatment groups.

Clinical characteristics	Total (*n* = 42)	HDPrT Group (*n* = 21)	HDPrT-C Group (*n* = 21)	Statistic	*P*
Sex, n (%)				*χ*^2^ = 0.10	0.757
Male	17 (40.48)	7 (33.33)	10 (47.62)		
Female	25 (59.52)	14 (66.67)	11 (52.38)		
Age, Mean ± SD	49.00 ± 8.07	48.00 ± 8.09	50.00 ± 8.12	t = 0.80	0.429
Pain Time (months) Mean ± SD	6.52 ± 1.86	6.43 ± 1.99	6.62 ± 1.77	t = 0.33	0.745
Affected side, n (%)				χ^2^ = 0.10	0.757
Left	19 (45.24)	9 (42.86)	10 (47.62)		
Right	23 (54.76)	12 (57.14)	11 (52.38)		
VAS, Mean ± SD	6.92 ± 0.63	6.76 ± 0.74	7.08 ± 0.46	t = 1.68	0.102
Tendon Thickness, Mean ± SD	6.00 ± 0.33	6.10 ± 0.35	5.90 ± 0.29	t = −2.01	0.051
Tendon Blood Supply, n (%)				χ^2^ = 0.38	0.537
Grade 0	NA	NA	NA		
Grade I	NA	NA	NA		
Grade II	20 (47.62)	9 (42.86)	11 (52.38)		
Grade III	22 (52.38)	12 (57.14)	10 (47.62)		

HDPrT, hypertonic dextrose prolotherapy; HDPrT-C, hypertonic dextrose prolotherapy combined with corticosteroid Injection; VAS, visual analogue scale scores; NA, not applicable.

No outliers were present in the dataset. VAS scores and tendon thickness data followed a normal distribution with homogeneity of variance. Tendon blood supply grade data did not conform to a normal distribution and exhibited heterogeneity of variance.

### Within-group comparisons of the two treatment protocols

Table 2 presents the differences in VAS scores, tendon thickness, and tendon blood supply grade at various follow-up intervals compared to baseline within each group.

**Table 2:** Comparison of VAS, tendon thickness and tendon blood supply between patients treated with HDPrT and HDPrT-C.

Parameter	Baseline	1 week	*adjusted* *P*	4 weeks	*adjusted* *P*	12 weeks	*adjusted* *P*	24 weeks	*adjusted* *P*
	Mean ± SD or n (%)	Mean ± SD or n (%)		Mean ± SD or n (%)		Mean ± SD or n (%)		Mean ± SD or n (%)	
VAS									
HDPrT	6.76 ± 0.74	6.70 ± 0.65	0.421	5.80 ± 0.68	<.001	3.80 ± 0.95	<.001	3.20 ± 0.65	<.001
HDPrT-C	7.08 ± 0.46	2.50 ± 0.69	<.001	1.80 ± 0.73	<.001	4.00 ± 1.38	<.001	2.90 ± 1.26	<.001
Tendon Thickness									
HDPrT	6.10 ± 0.35	6.10 ± 0.25	1.000	5.80 ± 0.30	0.002	5.50 ± 0.31	<.001	5.10 ± 0.20	<.001
HDPrT-C	5.90 ± 0.29	5.50 ± 0.25	<.001	5.60 ± 0.27	0.002	5.70 ± 0.30	0.039	4.80 ± 0.32	<.001
Tendon Blood Supply									
HDPrT			0.157		<.001		<.001		<.001
Grade 0	NA	NA		NA		NA		NA	
Grade 1	NA	2 (9.52)		8 (38.10)		14 (66.67)		17 (80.95)	
Grade 2	9 (42.86)	14 (66.67)		12 (57.14)		7 (33.33)		4 (19.05)	
Grade 3	12 (57.14)	5 (23.81)		1 (4.76)		NA		NA	
HDPrT-C			0.005		<.001		0.172		<.001
Grade 0	NA	NA		NA		NA		1 (4.76)	
Grade 1	NA	7 (33.33)		14 (66.67)		6 (28.57)		9 (42.86)	
Grade 2	11 (52.38)	13 (61.90)		7 (33.33)		7 (33.33)		10 (47.62)	
Grade 3	10 (47.62)	1 (4.76)		NA		8 (38.10)		1 (4.76)	

HDPrT, hypertonic dextrose prolotherapy; HDPrT-C, hypertonic dextrose prolotherapy combined with corticosteroid injection; VAS, visual analogue scale scores; NA, not applicable.

In the HDPrT group, compared to baseline, the change in VAS score at one week was not statistically significant (adjusted *P* = 0.421), whereas significant differences were observed at the 4 weeks, 12 weeks, and 24 weeks follow-up (all adjusted *P* < 0.001). In the HDPrT-C group, VAS scores showed statistically significant improvements from baseline at all follow-up time points: 1 week, 4 weeks, 12 weeks, and 24 weeks (all adjusted *P* < 0.001).

A significant interaction effect was found between group and time (F = 15.220, *P* < 0.001), prompting simple effects analysis. The simple effect of time was significant within both the HDPrT group (F = 81.456, *P* < 0.001) and the HDPrT-C group (F = 446.166, *P* < 0.001), indicating significant VAS score changes over time within each group.

Regarding tendon thickness, no significant change from baseline was detected in the HDPrT group at 1 week follow-up (adjusted *P* = 1.000). However, significant reductions were observed at 4 weeks, 12 weeks, and 24 weeks assessments (all adjusted *P* < 0.012). In the HDPrT-C group, no significant change from baseline was detected at 12 weeks follow-up (adjusted *P* = 0.039), but tendon thickness decreased significantly from baseline at 1 week, 4 weeks and 24 weeks follow-up (all adjusted *P* < 0.012). The simple effect of time on tendon thickness was significant within both the HDPrT group (F = 53.227, *P* < 0.001) and the HDPrT-C group (F = 65.902, *P* < 0.001).

A significant overall difference in tendon blood supply grade across time points was found in both the HDPrT group (*χ*^2^ = 33.384, *P* < 0.001) and the HDPrT-C group (*χ*^2^ = 54.547, *P* < 0.001). *Post-hoc* analysis for the HDPrT group revealed no significant change from baseline at 1 week (adjusted *P* = 0.157), but significant improvements at 4 weeks, 12 weeks, and 24 weeks (all adjusted *P* < 0.012). For the HDPrT-C group, no significant change from baseline was observed at the 12 weeks follow-up (adjusted *P* = 0.172), while significant improvements were noted at 1 week, 4 weeks, and 24 weeks (all adjusted *P* < 0.012).

### Between-group comparisons of the two treatment protocols

Figure 2 illustrates the changes in VAS scores for both groups during the follow-up period.

**Figure 2:**
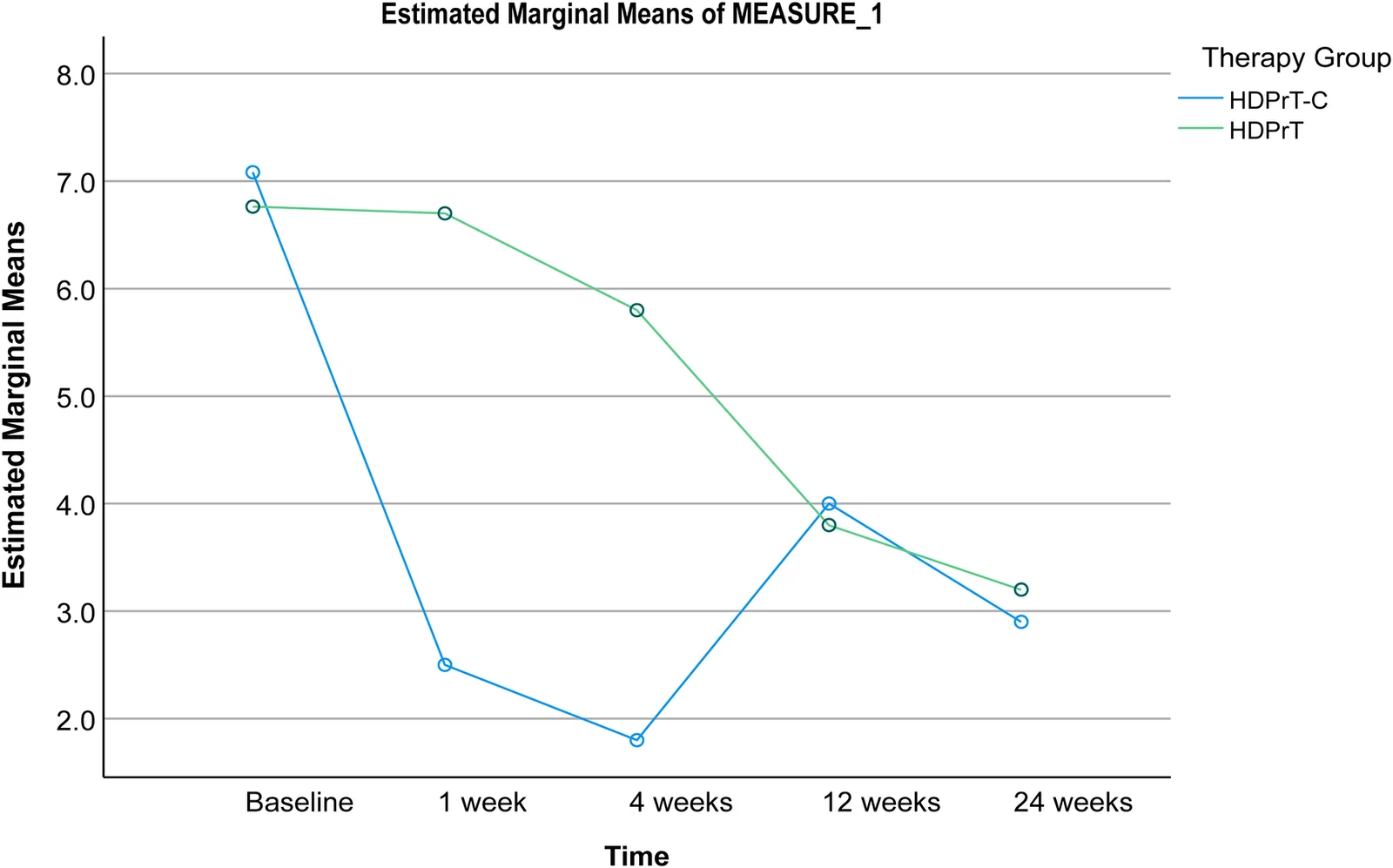
Representation of reduction of VAS following HDPrT-C versus HDPrT. HDPrT: Hypertonic Dextrose Prolotherapy; HDPrT-C: Hypertonic Dextrose Prolotherapy Combined with Corticosteroid Injection; VAS: Visual Analogue Scale Scores.

The simple effect of group analysis for VAS score reduction revealed a statistically significant difference between the HDPrT and HDPrT-C groups at 1 week (F = 407.077, *P* < 0.001) and 4 weeks (F = 341.811, *P* < 0.001) follow-up. No significant between-group difference was observed at 12 weeks (F = 0.299, *P* = 0.587) and 24 weeks (F = 0.945, *P* = 0.337) follow-up. *Post-hoc* tests revealed significantly lower VAS scores in the HDPrT-C group relative to the HDPrT group at 1 week (mean difference, −4.20 points; 95%CI −4.62 to −3.78, *P* < 0.001) and 4 weeks (mean difference, −4.00 points; 95%CI −4.44 to −3.56, *P* < 0.001) follow-up.

Figure 3 illustrates the changes in tendon thickness for both groups during the follow-up period.

**Figure 3:**
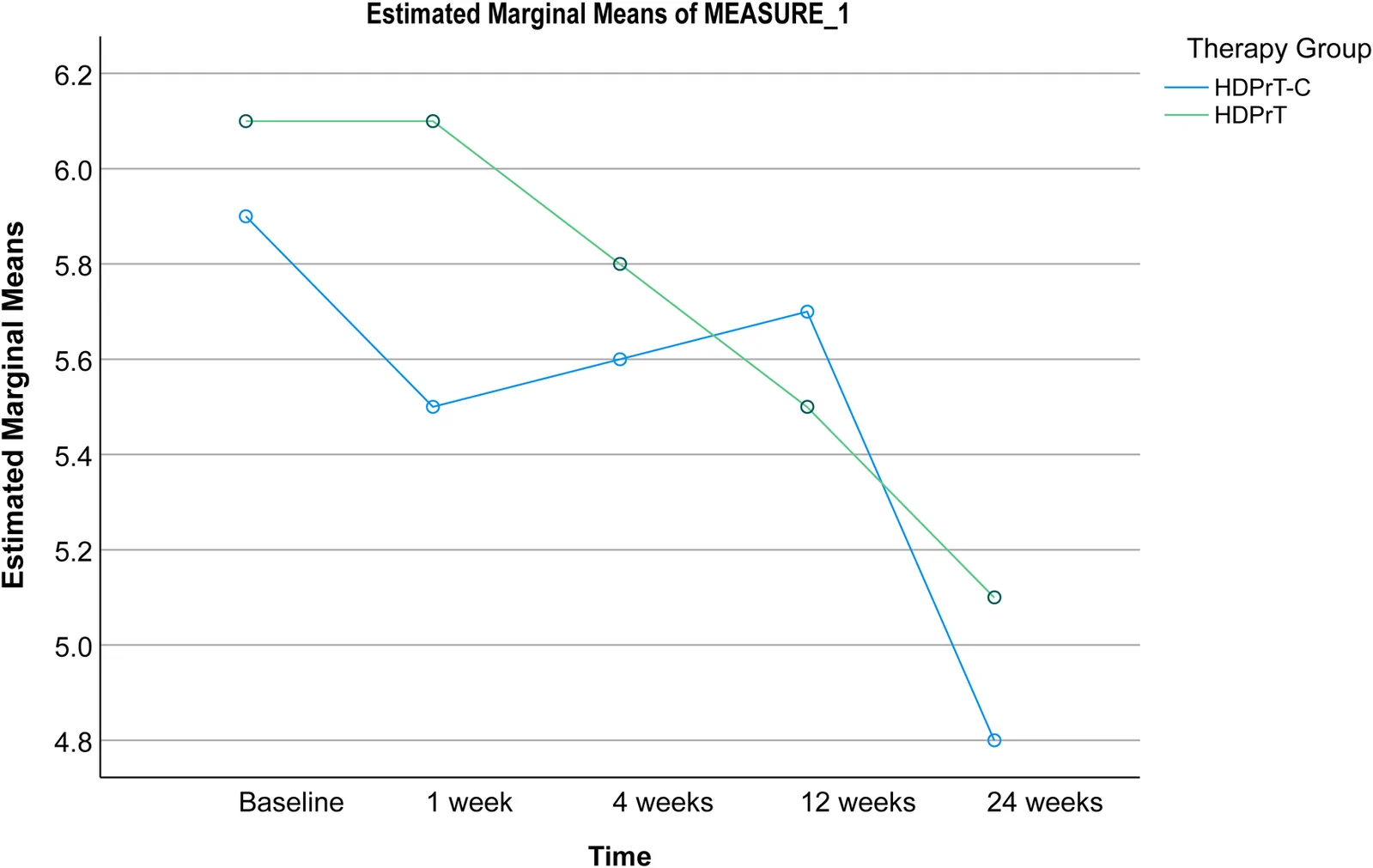
Representation of reduction of tendon thickness following HDPrT-C versus HDPrT. HDPrT: Hypertonic Dextrose Prolotherapy; HDPrT-C: Hypertonic Dextrose Prolotherapy Combined with Corticosteroid Injection.

For the reduction in tendon thickness, a statistically significant between-group difference was observed at all follow-up time points: 1 week (F = 60.000, *P* < 0.001), 4 weeks (F = 5.000, *P* = 0.031), 12 weeks (F = 4.492, *P* = 0.040), and 24 weeks (F = 13.034, *P* < 0.001). *Post-hoc* tests revealed significantly greater reductions in tendon thickness in the HDPrT-C group versus the HDPrT group: mean differences of −0.60 mm (95%CI −0.76 to −0.44) at 1 week, −0.20 mm (95%CI −0.38 to −0.19) at 4 weeks, and −0.30 mm (95%CI −0.47 to −0.13) at 24 weeks (all *P* < 0.05). Conversely, at 12 weeks follow-up, *post-hoc* tests revealed significantly smaller reductions in tendon thickness in the HDPrT-C group versus the HDPrT group: mean differences of 0.2 mm (95%CI: 0.09–0.39, *P* < 0.05).

The Mann–Whitney U test showed that no statistically significant difference in tendon blood supply grade was found between the two groups at the 1 week (U = 199.500, *P* = 0.542) or 4 weeks (U = 154.500, *P* = 0.056) follow-ups. However, significant differences were observed at the 12 weeks (U = 108.500, *P* = 0.002) and 24 weeks (U = 63.500, *P* = 0.048) follow-up.

## Discussion

This study found that HDPrT-C significantly reduced the VAS score and tendon thickness at short-term follow-up (1 and 4 weeks) compared with HDPrT, while no statistically significant intergroup difference was observed in the improvement of tendon blood supply grade. At medium- (12 weeks) and long-term (24 weeks) follow-up, HDPrT-C yielded reductions in tendon thickness and improvements in tendon blood supply grade, with no significant between-group difference in VAS scores. No complications were reported in either patient group following treatment.

HDPrT proved effective for LE management across all evaluated timeframes. Rabago et al. demonstrated significant improvements in Patient-Rated Tennis Elbow Evaluation and grip strength scores at 4, 8, and 16 weeks compared to a control group. Similarly, a meta-analysis by Zhu et al. concluded that HDPrT significantly improved pain and functional outcomes at 12 weeks, supporting its role as an effective non-surgical intervention (11). In our study, the HDPrT group exhibited progressive and significant reductions in VAS scores, tendon thickness, and tendon blood supply from baseline at 4 weeks, 12 weeks, and 24 weeks. The therapeutic mechanism was likely the initiation of a wound-healing cascade. *In vitro* research suggested that exposure to hypertonic dextrose triggered an inflammatory response in cells, characterized by the upregulation of pro-inflammatory markers such as interleukin-8, cyclooxygenase-2, and prostaglandin E2, alongside the downregulation of the anti-inflammatory marker transforming growth factor-beta (13).

The HDPrT-C was also effective for LE. The protocol involved peritendinous injection of betamethasone and intratendinous injection of hypertonic dextrose using a multi-point technique, which yielded significant therapeutic results. The combination group showed significant improvements in VAS scores, tendon thickness, and blood supply from baseline at all time points. Interestingly, we observed a slight rebound increase in these parameters at the 12 weeks follow-up after a sharp initial decline. This transient phenomenon was noted during clinical follow up interviews, in which some patients self-reported premature resumption of high-load elbow activities after experiencing marked short-term symptomatic relief between the 4-week and 12-week visits. Notably, activity-related exposure was not systematically recorded in this retrospective study. Therefore, the association between early return to high-load activity and this 12-week rebound remained a hypothesis. After being advised to modify activity during the 12 weeks assessment, these parameters showed improvement again by the 24 weeks follow-up.

The HDPrT-C group demonstrated superior short-term efficacy. Corticosteroid injection was used for LE for over six decades, but its long-term efficacy was suboptimal and associated with an increased risk of tendon atrophy or rupture. Lebiedziński et al. confirmed that betamethasone provided rapid onset and superior short-term outcomes (18). Conversely, Gupta et al. found HDPrT to be superior and more durable than HDPrT-C for LE (19). Our study suggested that the HDPrT-C offered superior short-term pain relief, while no statistically significant intergroup difference was observed at medium- and long-term follow-up compared to HDPrT. The peritendinous, rather than intratendinous, administration of corticosteroids in our protocol might help mitigate the iatrogenic risk of tendon rupture. Crucially, our results indicated that HDPrT-C yielded superior short-term pain relief relative to HDPrT. Although between-group differences in tendon thickness and tendon blood-supply grade were noted at medium-term (12-week) and long-term (24-week) follow-up, these exploratory findings cannot be considered definitive evidence supporting superior medium- to long-term efficacy of HDPrT-C.

This study has several limitations. First, the small sample size precluded meaningful subgroup analyses. Future research with larger cohorts could stratify patients by factors such as gender and age to analyze potential differential treatment responses. Second, the follow-up period was limited to 24 weeks. Longer-term observation, extending to one year or more, is necessary to fully evaluate the sustained effects of both treatment strategies. Third, this study relied on pain-related VAS scores and ultrasound morphological markers as outcome measurements, without including validated patient-reported functional scales such as Patient-Rated Tennis Elbow Evaluation (PRTEE), or objective physical measures such as grip strength. The effect of HDPrT-C on over functional improvement required further evaluation. Fourth, increased neovascularization was histopathologically linked to tendinopathy, but doppler vascularity grade did not always closely correlate with patient-reported pain and functional status. This ordinal grading scale was operator-dependent, and reduction in Doppler vascularity should not be equated directly with complete clinical recovery. Fifth, intra- and inter-observer reliability was not evaluated, which may affect the reproducibility of outcome measurements. Furthermore, no power calculation was performed, given the retrospective nature of this study. Sample size was determined by the number of eligible patients treated within the predefined time window. The limited sample size, repeated assessments and multiple pairwise statistical comparisons increased the risk of Type-I error (false-positive findings) and Type-II error (false-negative findings), meaning that genuine between-group differences at medium- and long-term follow-up might not be detected. Accordingly, our findings should be regarded as exploratory rather than definitive. Larger-scale, longer-duration randomized controlled trials are warranted to further assess the comparative therapeutic profiles of these two regimens.

## Conclusion

This exploratory study found that HDPrT-C offered superior short-term pain relief and greater reduction of tendon thickness in patients with LE. Observed medium- and long-term intergroup differences in tendon thickness and tendon blood supply grade were exploratory findings. Definitive evidence regarding medium- and long-term comparative efficacy required further investigation in larger-scale prospective or randomised controlled trials.

## Data Availability

The raw data supporting the conclusions of this article will be made available by the authors, without undue reservation.
